# Experiment and Theory in Concert To Unravel the Remarkable Electronic Properties of Na-Doped Eu_11_Zn_4_Sn_2_As_12_: A Layered Zintl Phase

**DOI:** 10.1021/acs.chemmater.3c01509

**Published:** 2023-09-14

**Authors:** Ashlee K. Hauble, Michael Y. Toriyama, Stephan Bartling, Ali M. Abdel-Mageed, G. Jeffrey Snyder, Susan M. Kauzlarich

**Affiliations:** † Department of Chemistry, University of California, One Shields Avenue, Davis, California 95616, United States; ‡ Department of Materials Science and Engineering, Northwestern University, Evanston, Illinois 60208, United States; § Leibniz Institute for Catalysis (LIKAT), Rostock 18059, Germany

## Abstract

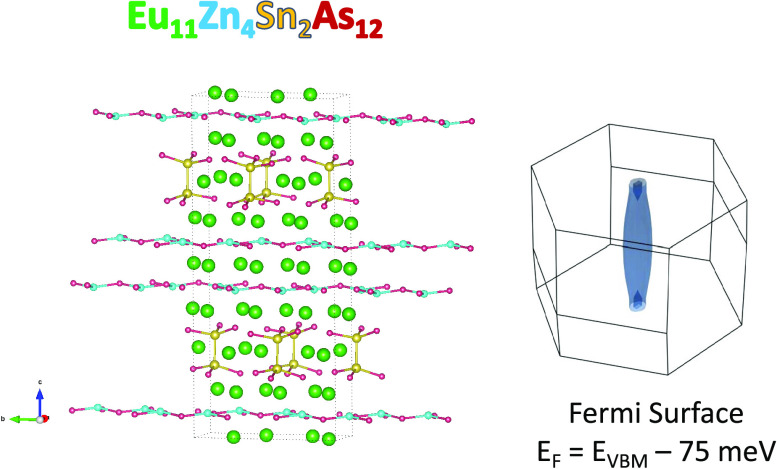

Low-dimensional materials have unique optical, electronic, mechanical, and chemical properties that make them desirable for a wide range of applications. Nano-scaling materials to confine transport in at least one direction is a common method of designing materials with low-dimensional electronic structures. However, bulk materials give rise to low-dimensional electronic structures when bonding is highly anisotropic. Layered Zintl phases are excellent candidates for investigation due to their directional bonding, structural variety, and tunability. However, the complexity of the structure and composition of many layered Zintl phases poses a challenge for producing phase-pure bulk samples to characterize. Eu_11_Zn_4_Sn_2_As_12_ is a layered Zintl phase of significant complexity that is of interest for its magnetic, electronic, and thermoelectric properties. To prepare phase-pure Eu_11–*x*_Na_*x*_Zn_4_Sn_2_As_12_, a binary EuAs phase was employed as a precursor, along with NaH. Experimental measurements reveal low thermal conductivity and a high Seebeck coefficient, while theoretical electronic structure calculations reveal a transition from a 3D to 2D electronic structure with increasing carrier concentration. Simulated thermoelectric properties also indicate anisotropic transport, and thermoelectric property measurements confirm the nonparabolicity of the relevant bands near the Fermi energy. Thermoelectric efficiency is known to improve as the dimensionality of the electronic structure is decreased, making this a promising material for further optimization and opening the door to further exploitation of layered Zintl phases with low-dimensional electronic structures for thermoelectric applications.

## Introduction

**Figure 1:**
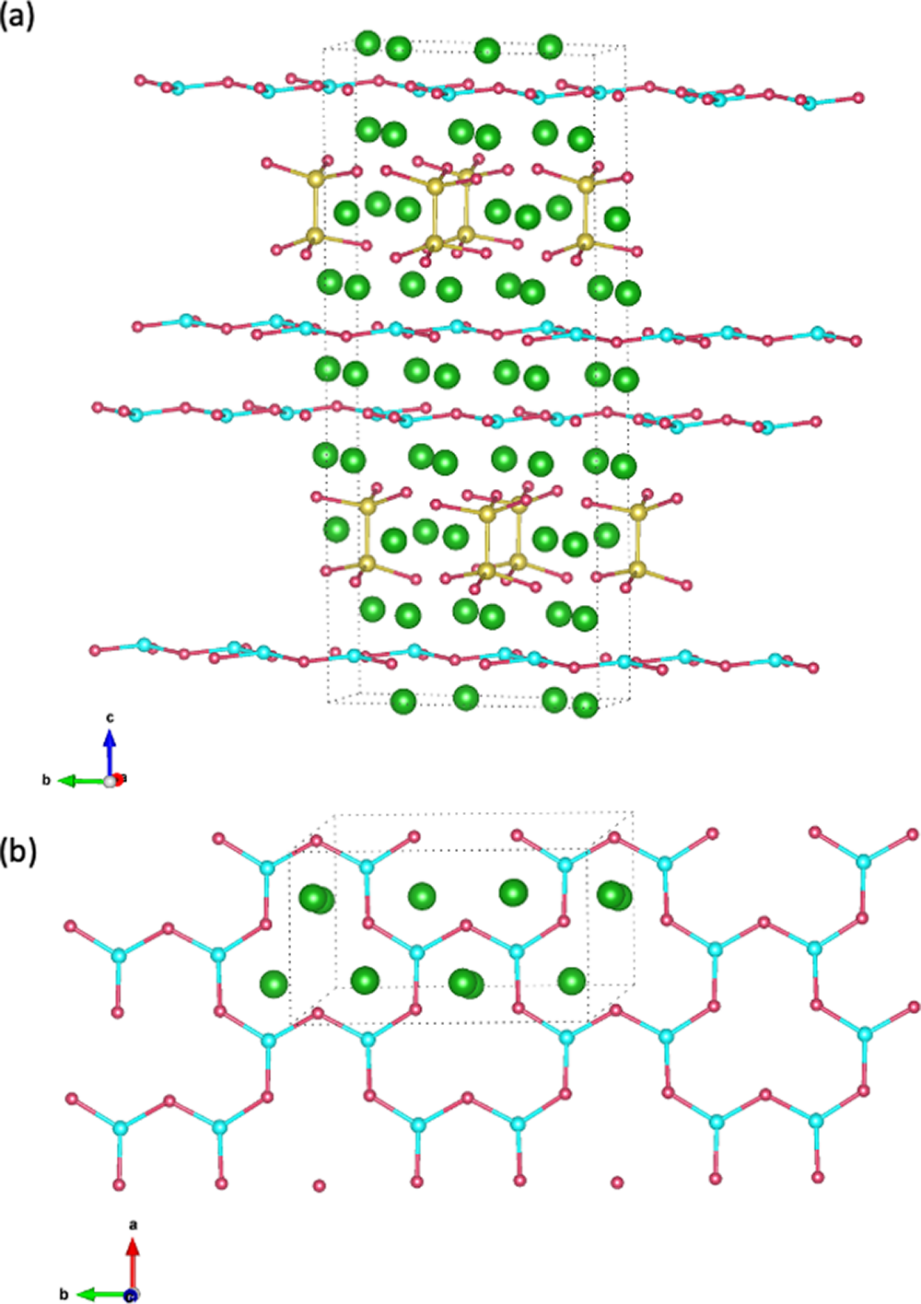
View of the crystal structure of Eu_11_Zn_4_Sn_2_As_12_ (*C*2/*c* space group) showing (a) layered aspect of the structure and (b) [Zn_2_As_3_]^5–^ defect honeycomb sheets. Eu atoms are indicated in green, Zn in turquoise, Sn in yellow, and As in red.

Layered materials with low-dimensional electronic structures are of interest for a wide variety of applications, including catalysis, optoelectronics, and energy harvesting.^1−5^ While low-dimensional electronic structures are generally achieved by nano-scaling materials to confine electronic transport in one or two directions, 3D structures can give rise to 1 or 2D electronic structures in bulk samples when bonding is highly directional.^6−9^ Layered materials are well-suited for this, given their anisotropic bonding. Much of the current research on layered materials is focused on 2D graphene and transition metal chalcogenides. However, Zintl phases with layered structures are a vast and underexplored class of materials with directional bonding and great structural variety, making them excellent candidates for the quest to uncover low-dimensional electronic structures in bulk semiconductors.^3,4,10,11^ The combination of ionic and covalent bonding in Zintl compounds gives rise to diverse bonding motifs that can be tuned to adjust the electronic structures, providing more opportunities to engineer low-dimensional transport. Several layered Zintl phases, including Yb_2–*x*_Eu_*x*_CdSb_2_, Eu_2_ZnSb_2_, and YbZn_2_Sb_2_, have already been shown to be promising thermoelectric materials and may find other applications (e.g., in catalysis^12^) if their electronic structures can be better understood.^13−18^ Eu_11_Zn_4_Sn_2_As_12_ is an example of a layered Zintl phase with complex, directional bonding (Figure 1), a commensurately modulated structure with crystallographic disorder that has the potential for a wide variety of uses.^19,20^

The crystal structure of Eu_11_Zn_4_Sn_2_As_12_ is shown in Figure 1. It is composed of layers of Eu cations, [Zn_2_As_3_]^5–^ defect honeycomb sheets, and pillars of ethane-like [Sn_2_As_6_]^12–^ units with interspersed Eu atoms.^19^ The delocalized bonding in the hexagonal [Zn_2_As_3_]^5–^ sheets, similar to the ZnSb_2_ layers found in the high-performing thermoelectric material Eu_2_ZnSb_2_, is expected to give rise to high charge carrier mobility in the *a*- and *b*-directions. In contrast, the ionic bonding along the *c*-direction is expected to contribute to high effective mass and low carrier mobility. In addition, the ethane-like [Sn_2_As_6_]^12–^ units should provide weak dispersion of the wave functions associated with their respective layer, further enhancing the 2D-like nature of this compound. These features result in a highly anisotropic and potentially 2D Fermi surface. In addition to high charge carrier mobility, delocalized bonding in the hexagonal nets can also give rise to topologically nontrivial electronic structures.^21^ A study on Eu_2_ZnSb_2_ revealed topological electronic structure changes based on vacancy ordering at the Zn site that are driven by the same physics as topological edge states in honeycomb-like nanoribbons.^22−25^ Complex, nonparabolic band structures, such as those present in topological materials, are often associated with high thermoelectric performance^26^ and are useful for many other purposes, such as spintronics, quantum information science, and catalysis.^6,8,21,27−29^ The known magnetic ordering and colossal magnetoresistance of Eu_11_Zn_4_Sn_2_As_12_ combined with the potential for topological behavior make this material an exciting candidate for a wide variety of applications.^19,20^ The crystal structure and magnetic properties of a related phase, Eu_3−δ_Zn_*x*_Sn_*y*_As_3_, were recently reported as a tunable structure for studying topological and magnetic properties.^30^

Given the vast potential for Eu_11_Zn_4_Sn_2_As_12_ and related phases, characterization of their electronic properties is necessary. However, as with many Eu-based compounds and complex structures, overcoming synthetic challenges to prepare phase-pure powders is a major consideration. Eu is malleable, reactive, and has high vapor pressure (3–4 times higher than that of other rare earth metals, not including Yb), making traditional solid-state synthesis routes difficult, particularly for Zintl phases where many different compounds exist with the same elements and similar stoichiometry (e.g., 2-1-2, 9-4-9, 11-6-12, 5-2-6, etc.).^10,31^ This work utilizes a binary synthesis route that provides excellent stoichiometric control in Yb_14_*M*Sb_11_ (*M* = Mn, Mg, Zn, Al).^32^ High-quality polycrystalline powders of Eu_11_Zn_4_Sn_2_As_12_ are obtained by employing a EuAs precursor that eliminates side phases that arise from Eu loss and insufficient mixing during synthesis. Using Eu binary compounds as synthetic precursors could unlock a multitude of compounds with novel properties, as Eu-based compounds are known to exhibit ferromagnetism, superconductivity, Kondo behavior, zero thermal expansion, high thermoelectric efficiency, and more, but an exploration of their properties has been limited by synthetic challenges.^10,14,15,19,20,31^

Here, we present the synthesis of phase pure Eu_11_Zn_4_Sn_2_As_12_ and dope the compound with Na to control carrier concentration. We measure thermoelectric properties and employ density functional theory (DFT) calculations to probe the low-dimensional electronic structure of Na-doped Eu_11_Zn_4_Sn_2_As_12_.

## Experimental Section

### Synthesis

Synthesis of polycrystalline samples of Eu_11–*x*_Na_*x*_Zn_4_Sn_2_As_12_ from stoichiometric amounts of the elements or the elements plus EuH_2_ leads to mixed phases with unidentified impurities. Therefore, polycrystalline Eu_11–*x*_Na_*x*_Zn_4_Sn_2_As_12_ (*x* = 0, 0.05, 0.075, 0.1) samples were synthesized via a balanced reaction using EuAs (ICSD-26265, the Na_2_O_2_ structure type)^33^ as a reactive precursor (synthesis described below) with NaH and the elements mixed via high-energy ball milling followed by annealing. In an Ar-filled glovebox, EuAs were combined with NaH powder (Aldrich, 90%), Zn flakes (Alfa, 99.98%), Sn shot (Alpha Aesar, 99.99%), and As lump (Johnson Matthey, Chemicals, 99.9999%) in a 65 mL stainless steel ball mill vial with two 12.7 mm diameter stainless steel balls. The vial was sealed in a mylar bag and placed in a SPEX 8000 M mill for 30 min, transferred to a glovebox to be scraped with a stainless-steel spatula, and then milled for an additional 30 min to ensure homogenization. The vial was scraped, and the powder was loaded into a 7 cm Ta tube that was sealed in an Ar-filled arc welder. The Ta tube was placed inside a fused silica tube, evacuated to <50 mTorr, flame sealed, and placed in a box furnace to be annealed at 800 °C for 96 h. The heating rate was 200 °C/h. The product was confirmed to be phase pure by powder X-ray diffraction and Rietveld refinement.

The EuAs precursor (ICSD- 26265, the Na_2_O_2_ structure type)^33^ was synthesized from stoichiometric amounts of the elements via high-energy ball milling and annealing. In an Ar-filled glovebox, Eu metal (Stanford materials, 99.99%) was cut into pea-sized pieces and combined with As lumps in a stainless-steel ball mill, hermetically sealed in a mylar bag, and milled for 30 min. Then, the vial was rotated 180 degrees and milled for an additional 30 min before transferring to a glovebox to be scraped and loaded into a 7 cm long (OD 0.953 x wall 0.051 cm) Ta tube that was arc-welded and further sealed in an evacuated silica tube. The powder was heated to 800 °C at a rate of 200 °C/h and annealed for 12 h. The product was confirmed to be phase pure by powder X-ray diffraction and Rietveld refinement (see Supporting Information (SI), Figure S1 and Table S1).

### Spark Plasma Sintering (SPS)

The phase-pure powder of Eu_11–*x*_Na_*x*_Zn_4_Sn_2_As_12_ (*x* = 0, 0.05, 0.075, 0.1), confirmed via PXRD, was ground with an agate mortar and pestle and sieved (100 mesh) and placed in a 12.7 mm inner diameter graphite die in a glovebox. The die was placed in a Spark Plasma Sintering instrument (Dr. Sinter Jr., Fuji Electronic Industrial Co., LTD) to be consolidated. After evacuating to below 15 Pa, the chamber was refilled with Ar to ∼50,000 Pa. The die was heated to 645 °C in 8 min and dwelled for 10 min. At 400 °C, the pressure was slowly increased from 47 to 83 MPa. The geometric and Archimedes densities of the sintered pellets were >95% of the theoretical density. Samples were sliced with a diamond saw (Buehler Isomet) and polished into thin, flat disks (<1.3 mm thick) for thermoelectric measurements.

### Powder X-ray Diffraction (PXRD)

Pieces of the sintered pellets obtained from SPS were ground into fine powders using an agate mortar and pestle for additional PXRD analysis. One pellet was also measured to check for the possibility of a preferred orientation. A Bruker D8 Advance Eco diffractometer with Cu Kα radiation was used to collect PXRD data. The diffractometer operated at 40 kV and 25 mA from 2θ range 20–80° with step size 0.015° and a scan rate of 1 s per step. Samples were determined to be single-phase Eu_11–*x*_Na_*x*_Zn_4_Sn_2_As_12_ via Rietveld refinement employing the crystallographic information file (CIF) with the TOPAS5 software (provided in Supporting Information, Figures S2 and S3, Table S2).^34^

### Scanning Electron Microscopy (SEM) and Energy-Dispersive X-ray Spectroscopy (EDS)

Pieces of sintered pellets were mounted in epoxy pucks and polished using sandpaper and a polishing wheel (1 μm colloidal diamond suspension) for SEM/EDS analysis with a Thermo Fisher Quattro ESEM equipped with a Bruker Quantax EDX detector. An Everhart–Thornley detector was used to collect secondary electron images with 20 kV accelerating voltage, and an annular backscatter detector was used for Z-contrast to analyze composition. EDS data from 10 spots, analyzed with the Bruker ESpirit software package, are provided in Supporting Information, Table S3. Na content was below the detection level of the instrument (<0.3 atomic %), so elemental maps for Na were not acquired.

### X-ray Photoelectron Spectroscopy (XPS)

XPS measurements were carried out on an ESCALAB 220iXL (Thermo Fisher Scientific) spectrometer equipped with a monochromated Al Kα X-ray source (*E* = 1486.6 eV). The pellets were prepared on a stainless-steel holder with conductive double-sided adhesive carbon tape. Before survey and detailed analysis, samples were exposed to Argon sputtering with 2 kV at a pressure of 2 × 10^–7^ mbar Ar. Sputtering was carried out for 10 min and then for 180 min to ensure the removal of any contaminants from the surface. The electron binding energies were obtained without charge compensation, and no further referencing has been applied. For quantitative analysis, the peaks were deconvoluted with Gaussian-Lorentzian curves using the software Unifit 2023. The peak areas were normalized by the transmission function of the spectrometer and the element-specific sensitivity factor of Scofield.^35^

### Thermoelectric Properties

A Netzsch Laser Flash Analysis (LFA) 475 Microflash instrument was used to measure thermal diffusivity on sintered pellets under a flow of Ar (60 mL/min) from 300 to 675 K and back to down 350 K. Diffusivity was used to calculate thermal conductivity from the relationship κ = λρ*C*_p_, where κ is thermal conductivity, *C*_p_ is the Dulong-Petit heat capacity, λ is thermal diffusivity, and ρ is sample density. Hall effect and resistivity measurements were done at the Jet Propulsion Laboratory via the Van der Pauw method with a 100 mA current and a 1.0 T magnet. The instrumental setup is described elsewhere.^36^ Seebeck coefficients were measured at the Jet Propulsion Laboratory with a custom light pipe instrument via the two-probe method with tungsten-niobium thermocouples under high vacuum.^37^

### Electronic Structure Calculations

First-principles DFT calculations were performed using the Vienna ab initio simulation package^38,39^ under the projector-augmented wave formalism.^40,41^ The Perdew–Burke–Ernzerhof functional was used to describe the exchange–correlation interaction.^42^ An energy cutoff of 340 eV and the experimentally resolved crystal structure of Eu_11_Zn_4_Sn_2_As_12_ at 90 K^19^ were used in all calculations. The Eu-*f* electrons were treated as core electrons. Spin–orbit coupling (SOC) was included in all calculations.

The transport properties, namely, the electrical resistivity and Seebeck coefficient, were calculated from Onsager transport coefficients using the Ab initio Scattering and Transport (AMSET) software.^43^ We found that the transport properties are well-converged when the 6 × 6 × 2 *k*-point mesh is interpolated by a factor of 15.^44^ All required material parameters were calculated using DFT, namely, the finite difference method to obtain the stiffness tensor and density functional perturbation theory to obtain the dielectric tensor.

## Results and Discussion

### Sample Purity and Composition

The synthesis of phase-pure Eu_11–*x*_Na_*x*_Zn_4_Sn_2_As_12_ (*x* = 0, 0.05, 0.075, 0.1) via the elements or the elements and some amount of EuH_2_ was unsuccessful. We found that by replacing Eu metal or metal hydride with EuAs (ICSD-26265, the Na_2_O_2_ structure type, Supporting Information, Table S1 and Figure S1)^33^ as a reactive precursor produced phase-pure Eu_11–*x*_Na_*x*_Zn_4_Sn_2_As_12_ (*x* = 0, 0.05, 0.075, 0.1; Supporting Information, Figures S2 and S3, Table S2). Initially, samples were prepared for *x* = 0.05, 0.1, 0.2, 0.3, but samples with higher Na content (*x* = 0.2, 0.3) sublimed Na vapor when heated, suggesting *x* = 0.1 is the maximum amount of Na that could be incorporated into the structure. This reactive precursor may be applied to other Eu-based compounds to enable high-quality synthesis of complex phases.

Zintl–Klemm electron counting rules can be used to rationalize Eu_11_Zn_4_Sn_2_As_11_ as a charge-balanced semiconductor by considering the [Sn_2_As_6_]^12–^ units as Sn^0^ (four bonds) and As^2–^ (one bond), the [Zn_2_As_3_]^5–^ sheets as As^–^ (two bonds) and Zn^–^ (three bonds) and the cations as isolated Eu^2+^.^19^ The compound was initially described as a monoclinic structure in the space group *C2*/*c* with site disorder and a stacking perturbation.^19^ However, reinvestigation of the Eu_11_Zn_4_Sn_2_As_11_ structure showed that it is better described as a commensurately modulated structure in the *R*3̅*m* space group with a wave vector (*Q*) of 2/3, 2/3, 0.^20^ Upon substitution of Eu with Sr, the symmetry is reduced, and the structure is also described as a commensurately modulated structure in the *R*3̅ space group but with a modified wave vector of 1/3, 1/3, 1/2. The *C*2/*c* space group provides an average structure with disorder (shown in Figure 1 for simplicity and to emphasize the layered nature of the compound), but the rhombohedral space groups more accurately describe the modulation and partial site occupancy. In both rhombohedral space groups, the Zn and Sn sites have vacancies, and their site occupancies are 2/3 and 1/3, respectively. The vacancies within the [Zn_2_As_3_]^5–^ sheets give rise to substitutional disorder. In the layer containing isolated Sn_2_As_6_ ethane-like units, there is an additional substitutional disorder modeled by a Eu site situated along the Sn–Sn bond that is occupied 2/3 of the time, while the Sn sites are occupied 1/3 of the time. The more ionic and slightly larger Sr cation (ionic radii 1.18 Å) substitutes on the Eu sites between the [Zn_2_As_3_]^5–^ sheets and those within the [Sn_2_As_6_]^12–^ units and in a slightly shifted position relative to the Eu site that is found between the [Zn_2_As_3_]^5–^ sheets and the [Sn_2_As_6_]^12–^ units.^20^ The Na ion is smaller (ionic radii 1.02 Å), more electropositive than Eu (ionic radii, 2+, 1.17 Å), and could also exhibit site preference.

**Figure 2:**
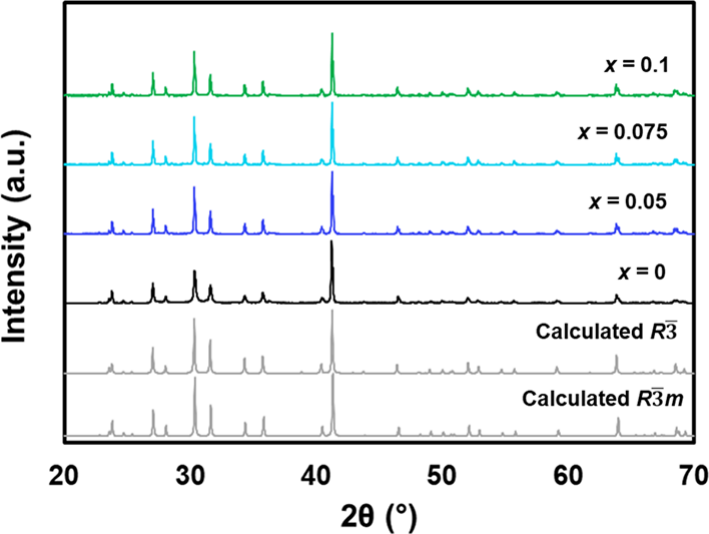
PXRD patterns for Eu_11–*x*_Na_*x*_Zn_4_Sn_2_As_12_ (*x* = 0, 0.05, 0.075, 0.1) compared to the calculated *R*3̅*m* pattern (bottom) and *R*3̅ (second from bottom).

PXRD patterns of ground pellets of Eu_11–*x*_Na_*x*_Zn_4_Sn_2_As_12_ (*x* = 0, 0.05, 0.075, 0.1) are compared to the calculated *R*3̅*m* and *R*3̅ patterns in Figure 2. The diffraction patterns for both space groups are nearly identical. The best refinement for the samples was obtained using the *R*3̅*m* CIF. The Na-doped samples were also refined employing the *R*3̅ CIF; however, the *R*_p_ and *wR*_p_ values were very similar for the two space groups. Rietveld refinement parameters are summarized in Table S2 with lattice parameters from the *R*3̅*m* space group and are consistent with phase pure Eu_11–*x*_Na_*x*_Zn_4_Sn_2_As_12_ for all compositions. A slight decrease in lattice parameters with Na was observed, consistent with the smaller size of Na compared to Eu. Because of the layered nature of the structure, we also checked for preferred orientation by collecting X-ray diffraction of the pressed pellet. The relative intensities of the experimental data were consistent with the calculated pattern, suggesting that no preferred orientation is present in the pellet (see Supporting Information, Figure S4).

**Figure 3:**
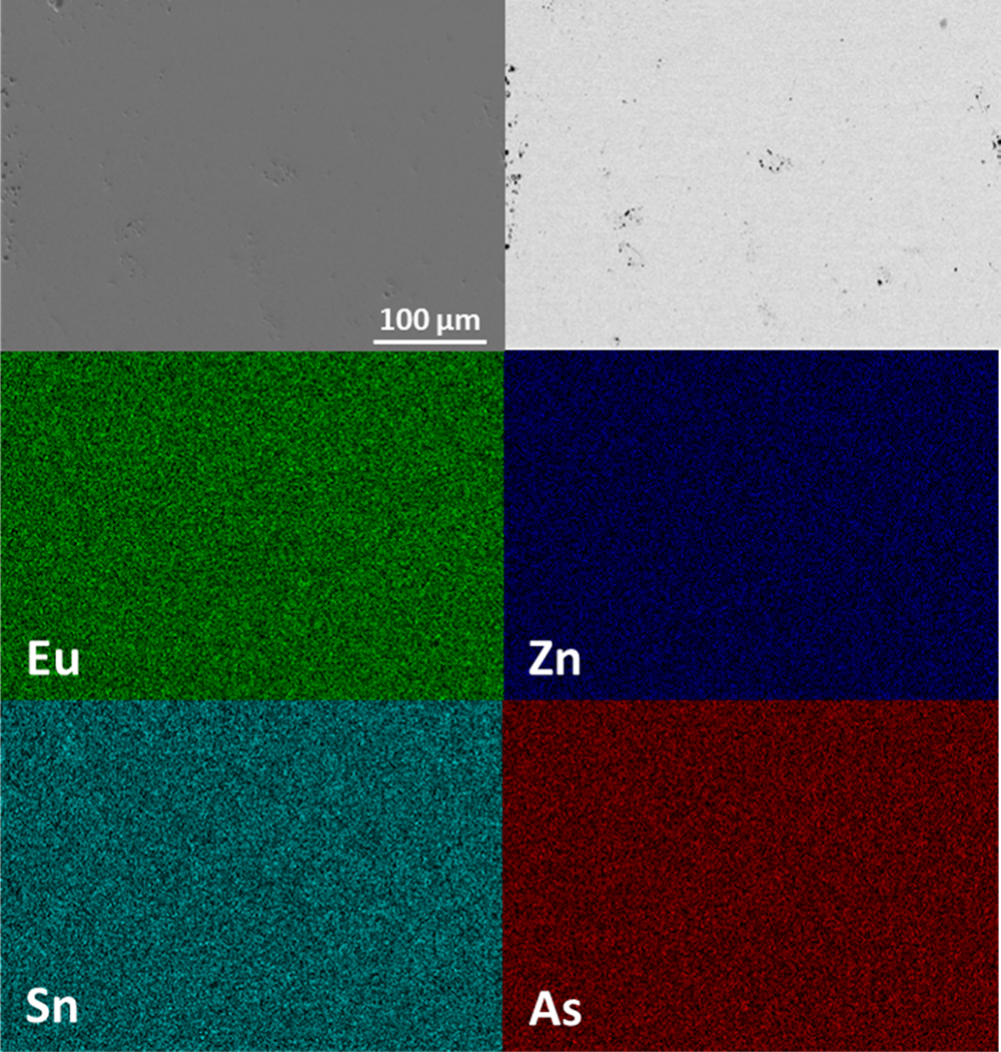
SEM micrographs from secondary electrons (top left) and backscattered electrons (top right) and EDS elemental maps (bottom) for all elements except Na (signal below background) for a pressed pellet of Eu_11–*x*_Na_*x*_Zn_4_Sn_2_As_12_ with the greatest Na content, *x* = 0.1.

Figure 3 shows the SEM micrographs and EDS elemental maps for the sample with the largest amount of Na (*x* = 0.1). The backscattered electron image shows that the sample is uniform, and there are no obvious pockets of a secondary phase. The elemental maps show the spatial distribution of the element of interest and indicate a homogeneous sample. The elemental map for Na was not acquired because the amount was below the detection limit (<0.3 atomic %). Results from energy-dispersive spectroscopy are provided in Supporting Information, Table S3 compared to their nominal compositions. All samples show atomic percent’s that are slightly Eu and As rich except for the *x* = 0.075 composition, which shows lower Eu and Zn and higher As than nominal. Secondary element SEM micrographs and EDS elemental maps for *x* = 0, 0.05, 0.075 are provided in Supporting Information, Figure S5. The samples show homogeneous distribution of elements with no obvious secondary phases.

### XPS

**Figure 4:**
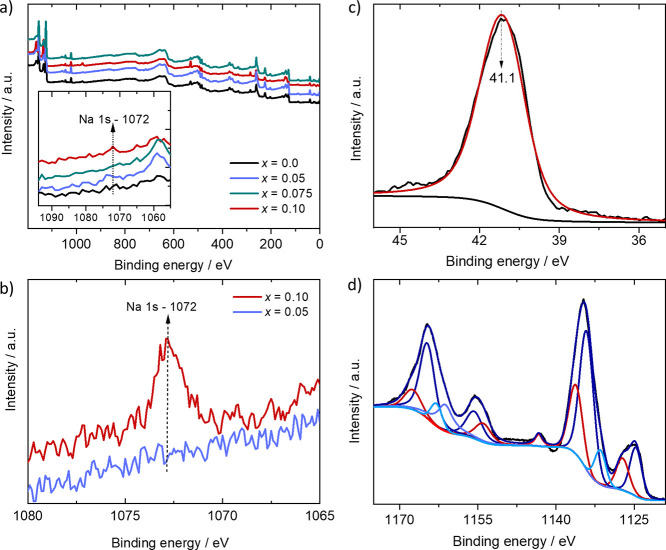
(a) Survey spectra for all samples after 10 min of Ar sputtering (inset focusing on the Na 1*s* region); (b) detailed scan of the Na 1*s* region (1065 to 1082 eV) for *x* = 0.1 and *x* = 0.05 samples. (c) As 3*d* and (d) the Eu 3*d* regions for the *x* = 0.1 Na-loaded sample.

XPS measurements were employed to examine Na content (Figure 4) in the prepared samples. A survey spectrum of the four samples after 10 min of Ar sputtering is displayed in Figure 4a. The Na 1*s* peak is visible only in the most heavily doped sample (*x* = 0.1) (see the inset in Figure 4a). The Na content in the lower *x*-value samples is below the instrument’s detection limit (0.3 atomic %). To confirm this result, we performed a detailed scan over the Na 1*s* region for all samples (Figure 4b). Detailed scans were also employed for Eu and As in the As 3*d* and the Eu 3*d* regions. We showed in Figure 4c,d the spectra of the sample with the highest Na concentration (see spectra of other samples in Supporting Information, Figures S6 and S7, Supporting Information). After 10 min of Ar sputtering, we observed in all samples the core level of As with a binding energy of 41.1 eV with insignificant shifts from one sample to another, indicating the presence of an anionic As species similar to what has been reported for ZrCuGeAs and BaAg_2_As_2_ (see Figure 4c and Supporting Information, Figure S6).^45,46^ In the Eu 3*d* region (Figure 4d), a rather complex peak structure, caused by final state effects, is observed. The main peaks around 1125 and 1134.5 eV can be assigned to Eu^2+^ and Eu^3+^, respectively.^47^ Each peak is fitted with a main component together with a shake-up peak. Additionally, Eu Auger peaks overlap with the Eu 3*d* region and lead to further peaks. The Eu 3*d* peak deconvolution shows a major contribution of Eu^2+^ ions compared to Eu^3+^ on all studied samples except in *x* = 0.1, which showed a significant Eu^3+^ signal intensity (see Figure 4d), roughly 3:1 ratio (Eu^3+^:Eu^2+^). The Eu^3+^ contribution in the *x* = 0.1 sample is likely due to a surface oxide layer promoted by a larger concentration of Na ions. To address this, samples were sputtered for 180 min and remeasured (Supporting Information, Figure S7). The additional sputtering time removed the majority of Eu^3+^ contribution, and the spectra of all samples under these conditions became similar. However, it also removed the Na 1*s* signal, suggesting that the amount of Na in the *x* = 0.1 sample is less than what was loaded, so it is more likely to be less than 0.1, but more than 0.075 and less than what can be measured (see discussion of carrier concentration below). Therefore, some Na is likely present at the grain boundaries for the *x* = 0.1 sample. Based on this observation, we tentatively attribute the coexistence of measurable concentration of Na with the promotion of Eu^2+^ oxidation to Eu^3+^ species. The disappearance of the Na signal upon longer sputtering periods suggests, however, that for *x* = 0.1, Na exists preferentially on the topmost layers (i.e., segregating to the surface) or at the grain boundaries.

### Thermal Transport Properties

**Figure 5:**
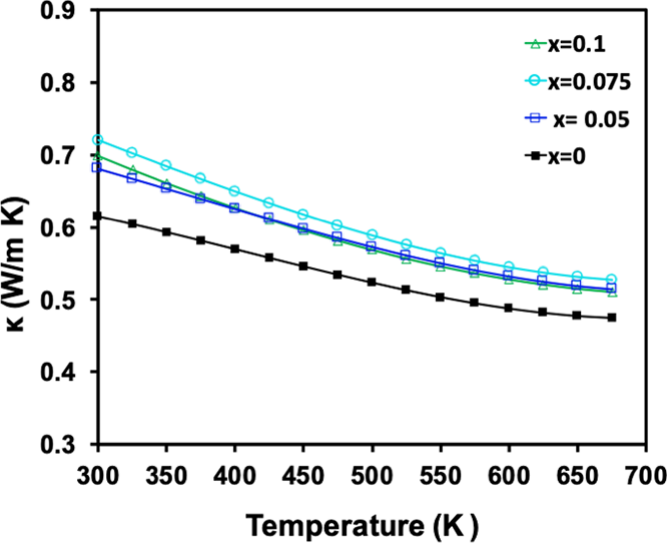
Temperature-dependent thermal conductivity from 300 to 675 K for Eu_11–*x*_Na_*x*_Zn_4_Sn_2_As_12_ (*x* = 0, 0.05, 0.075, 0.1).

Total thermal conductivity is shown in Figure 5 for Eu_11–*x*_Na_*x*_Zn_4_Sn_2_As_12_ (*x* = 0, 0.05, 0.075, 0.1) and is low as expected, given the complex structure and crystallographic disorder.^2,48,49^ As Na content increases, total thermal conductivity increases slightly, consistent with greater electronic contribution expected for doped samples, but remains lower than 0.8 W/m K at 300 K for all compositions and falls to ∼0.5 W/m K at 675 K. These values are lower than those reported for the related A_11_Cd_6_Pn_12_ systems and comparable to or lower than those reported for leading thermoelectric materials such as the Zintl phases Yb_14_MnSb_11_, Eu_2_ZnSb_2_, A_2_CdSb_2_, Yb_21_Mn_4_Sb_18_, and YbZn_2_Sb_2_ as well as PbTe and La_3–*x*_Te_4_.^13−16,49−53^ Because of the relatively low electrical conductivity, the thermal conductivity measured should be almost entirely due to phonon (lattice vibration) heat transport or lattice component to the thermal conductivity. The electronic contribution can be estimated from an estimate of the Lorenz factor^54,55^ and should be less than 1% of the measured value. The ultralow lattice thermal conductivity of Eu_11–*x*_Na_*x*_Zn_4_Sn_2_As_12_ can be attributed to the complex structure, likely to be dominated by diffuson rather than classical wavelike phonons.^56,57^

### Electronic Transport Properties

Experimental hole concentrations determined via the Hall effect for Eu_11–*x*_Na_*x*_Zn_4_Sn_2_As_12_ for *x* = 0.075, 0.1 are provided in Table 1 (values for *x* = 0 and 0.05 were <10^18^ and are provided in Supporting Information, Table S4). The carrier concentrations increase with Na, consistent with the expectation of replacing Eu^2+^ with Na^+^ in this p-type semiconductor. The relatively low starting carrier concentration suggests few intrinsic defects and is attributed to the precise stoichiometric control offered by the binary synthesis method.^32^ Peak *zTs* typically occur at carrier concentrations between 10^19^ and 10^20^ carriers per cm^3^, depending on the system; however, attempts to increase the number of carriers via Na-doping further were unsuccessful, and the solubility limit of Na was found to be *x* ≤ 0.1.

**Table 1:** Hall Carrier Concentration and Mobility of Eu_11–*x*_Na_*x*_Zn_4_Sn_2_As_12_ at 300 K

*x*	carrier concentration (cm^–3^)	Hall mobility (cm^2^/V s)
0.075	1.0 × 10^18^	10
0.1	1.1 × 10^18^	15

**Figure 6:**
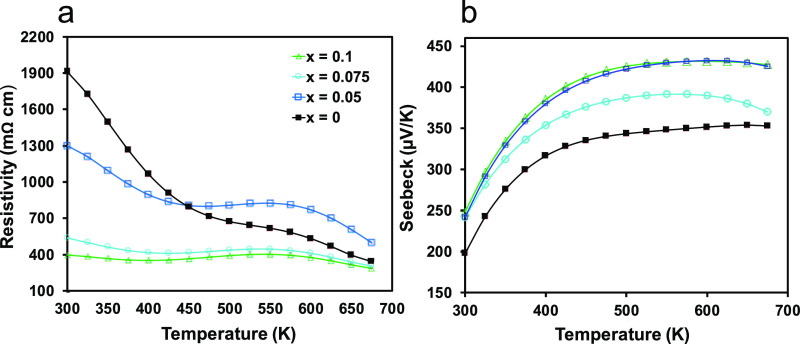
Temperature-dependent (a) electrical resistivity and (b) Seebeck coefficients from 300 to 675 K for Eu_11–*x*_Na_*x*_Zn_4_Sn_2_As_12_ (*x* = 0, 0.05, 0.075, 0.1). The legend is the same for both plots.

The observed increase in carrier concentration is accompanied by an increase in Hall mobility (Table 1), an anomalous result that could be due to changes in microstructure and leads to a nearly five-fold reduction in room temperature electrical resistivity with Na-doping, from ∼1900 mΩ cm for the undoped sample to ∼400 mΩ cm for *x* = 0.1 at 300 K (Figure 6a). The resistivity measurements show that Eu_11_Zn_4_Sn_2_As_12_ is a highly resistive semiconductor, as expected from Zintl–Klemm electron counting rules.

Seebeck coefficients (Figure 6b) are high and positive across the entire series (from ∼200 to ∼450 μV/K) and increase with temperature up to ∼600 K, giving a Goldsmid-Sharp band gap of 0.51 eV for *x* = 0 that agrees reasonably well with the calculated value of 0.6 eV (see the next section). The Seebeck coefficients increase as Na content increases, which is unexpected due to the inverse relationship between the Seebeck coefficient and carrier concentration in systems with a single dominant band.^49^ While this result could be due to microstructure effects, such as commensurate modulation, dislocations, grain boundaries, impurities, or Na site preference, which have been shown to dominate charge transport in a variety of materials,^58−60^ it could also be attributed to a rapidly changing effective mass with doping, which can be an indication of a highly anisotropic Fermi surface.^6,8,49^ In 2D electronic structures, the effective mass in the direction parallel to the length of the tube is infinite, limiting electronic transport to the two lighter directions.^6,7^ This can decouple the Seebeck coefficient and electrical resistivity effective masses because resistivity is dominated by the lightest mass direction, while the Seebeck coefficient is governed by the average effective mass, making it possible to increase the Seebeck coefficient without increasing electrical resistivity.^6^ This effect has been predicted to yield a high power factor in AZn_2_Sb_2_ Zintl phases (A = Ca, Sr, Ba),^61^ Ca_5_In_2_Sb_6_,^62^ Heusler compounds Fe_2_*YZ* (*Y* = Ti, Zr, Hf, and *Z* = Si, Ge, Sn),^7^ and lead chalcogenides.^8,9^

Parker et al. explain the increase in thermopower with decreasing dimensionality in terms of increasing Fermi surface volume.^9^ For a given *E*_F_, the carrier concentration (or volume of the Fermi surface) is larger in a 2D cylinder as compared to a 3D sphere because it is proportional to the length of the cylinder, which gives a much larger value than the radius of the cylinder or sphere. According to the Mott equation for the Seebeck coefficient, thermopower is inversely proportional to *E*_F_. For a given carrier concentration, *E*_F_ is smaller in 2D than in 3D, so the resulting thermopower is larger.^9^ As Eu_11–*x*_Na_*x*_Zn_4_Sn_2_As_12_ is more heavily doped, the Fermi surface should become increasingly cylindrical and 2D-like, leading to a higher surface area and greater thermopower for the same carrier concentration.

**Figure 7:**
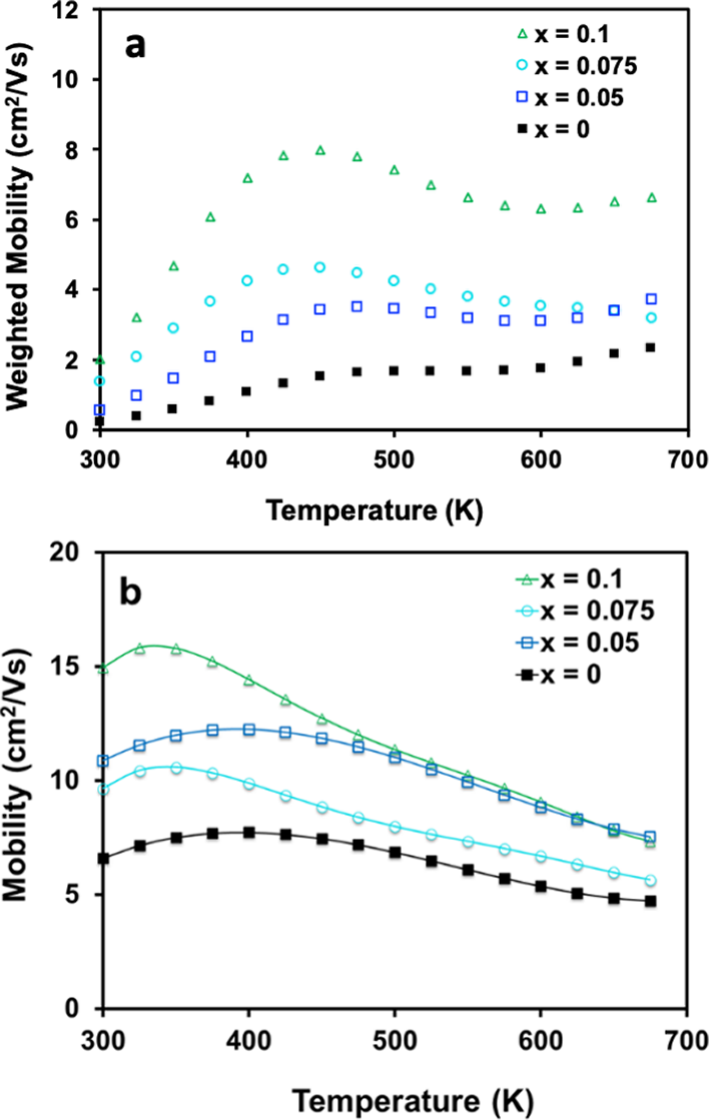
Temperature-dependent (a) weighted mobility and (b) Hall mobility for Eu_11–*x*_Na_*x*_Zn_4_Sn_2_As_12_ (*x* = 0, 0.05, 0.075, 0.1) from 300–675 K.

Weighted mobility and Hall mobility are shown in Figure 7. Weighted mobility is the charge carrier mobility weighted by the electronic density of states and is independent of charge carrier concentration. When charge transport is dominated by a single parabolic band and charge carrier concentration is changed via doping, weighted mobility values are expected to be the same for samples at different doping levels. Changing weighted mobility values can indicate nonparabolicity or multiband transport.^63^Figure 7a shows weighted mobility that increases with Na content, suggesting that carrier concentration is not the only variable changing. The weighted mobility increases rapidly with temperature from 300 to 450 K, while the Hall mobility given in Figure 7b displays thermally activated transport at low temperatures, consistent with grain boundary scattering. It is also important to note that the thermally activated Hall mobility is an indication that microstructure influences electronic transport in these samples. Temperature-dependent *zTs* are low due to the high resistivity with *zT*_max_ ∼ 0.1 achieved at 675 K for the *x* = 0.1 sample (*zT* vs *T* provided in Supporting Information, Figure S9).

### Calculated Electronic Structure and Transport Properties

**Figure 8:**
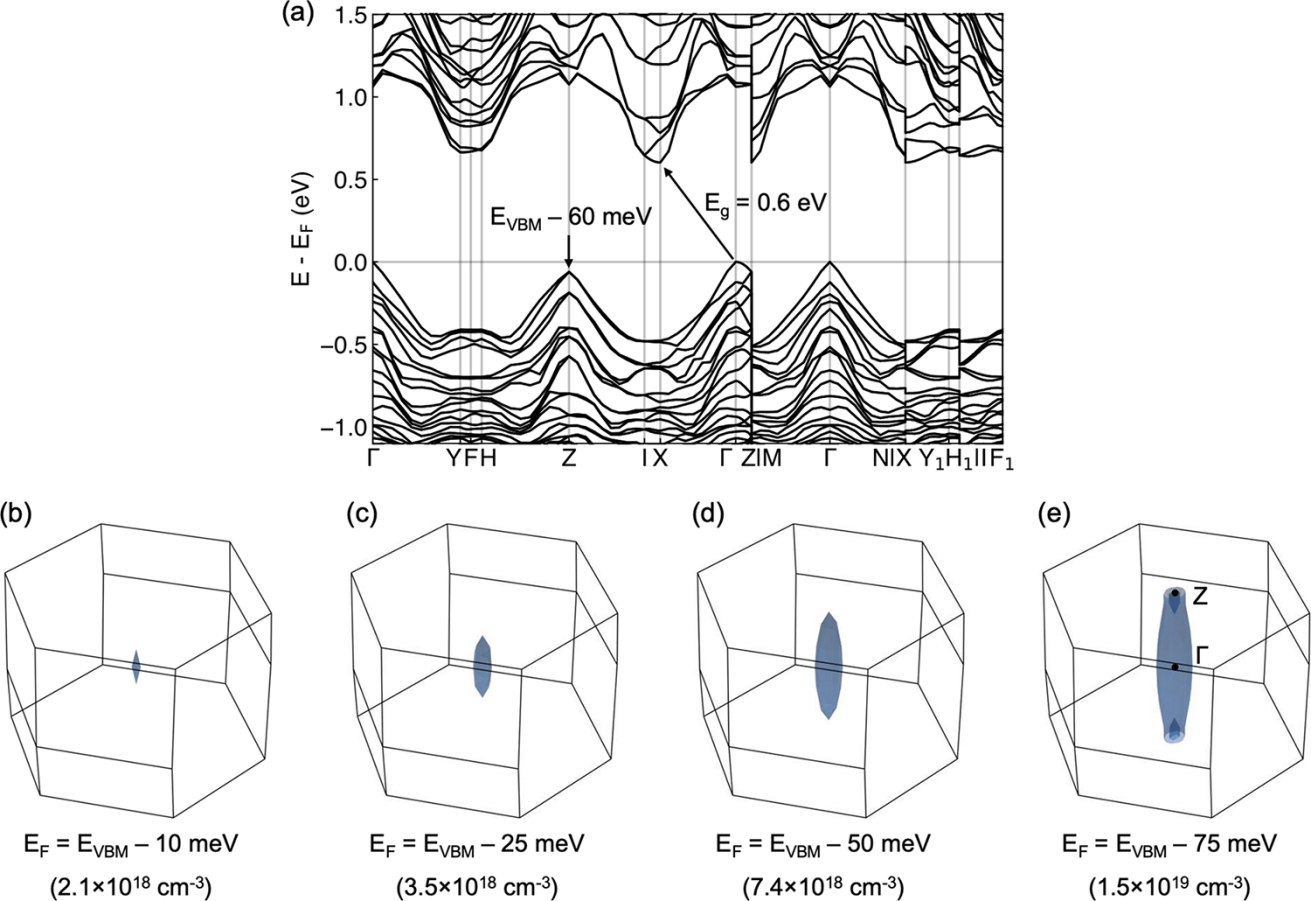
(a) Band structure of Eu_11_Zn_4_Sn_2_As_12_ calculated with spin–orbit coupling. An indirect band gap of *E*_g_ = 0.6 eV forms between the VBM at the Γ-point and the conduction band minimum at the X-point. A secondary VBM exists 60 meV below the VBM. (b–e) Fermi surfaces at the listed Fermi levels inside the valence band, along with the corresponding carrier concentrations at 300 K. The ellipsoidal Fermi surfaces suggest anisotropic electrical transport.

The electronic band structure of Eu_11_Zn_4_Sn_2_As_12_ and the corresponding Fermi surfaces at different Fermi levels are shown in Figure 8. Note that the band structure in Figure 8 is equivalent to that of Devlin et al.,^19^ except that a standard *k*-point path^64^ is used and spin–orbit coupling (SOC) is included in the calculation. An indirect band gap of 0.6 eV is calculated for Eu_11_Zn_4_Sn_2_As_12_, in good agreement with the Goldsmid–Sharp gap (∼0.51 eV) calculated from the experimental data. The ellipsoidal geometry of the Fermi surfaces near the valence band maximum (VBM) suggests that the transport properties are anisotropic, as expected from the monoclinic space group (*C2*/*c*) and the layered structure. In addition to the VBM at the Γ-point, the band structure and Fermi surface show a secondary valence band at the *Z*-point which is 60 meV below the VBM, indicating that band convergence may be a route toward optimizing the thermoelectric performance of Eu_11_Zn_4_Sn_2_As_12_.

**Figure 9:**
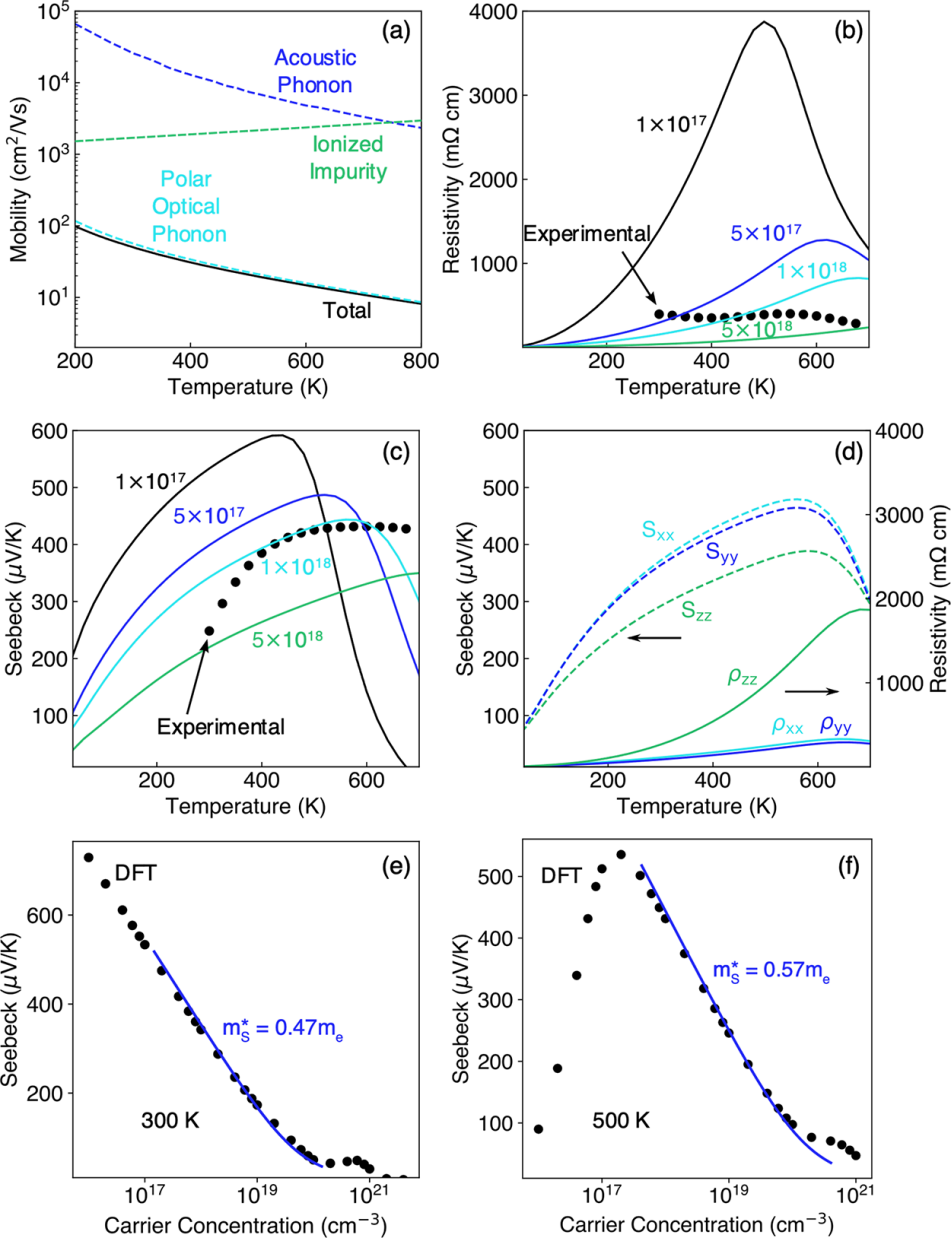
(a) Predicted hole mobility in Eu_11_Zn_4_Sn_2_As_12_ at a hole concentration of 1 × 10^18^ cm^–3^, where scattering by acoustic phonons (blue), ionized impurities (green), and polar optical phonons (cyan) is shown. The predicted temperature-dependent electrical resistivity (b) and Seebeck coefficient (c) are shown for different hole concentrations. The transport properties are averaged over the three Cartesian directions in (b, c), and the direction components are shown in (d) at a carrier concentration of 1 × 10^18^ cm^–3^. Pisarenko relations at 300 K (e) and 500 K (f) are used to compute the hole effective mass, calculated using the formula in ref (67).

To understand the transport properties of pristine Eu_11_Zn_4_Sn_2_As_12_, detailed Boltzmann transport theory-based calculations were run. Since the Eu_11_Zn_4_Sn_2_As_12_ samples synthesized in the present study display a p-type character, the properties of hole transport using DFT were investigated. The effects of deformation potential scattering by acoustic phonons, inelastic polar optical phonon scattering, and scattering by ionized impurities on charge carrier transport are addressed, as opposed to assuming a constant relaxation time.^65,66^ The effects of the three scattering mechanisms on the hole mobility are shown in Figure 9a, indicating that polar optical phonons limit hole transport in p-type Eu_11_Zn_4_Sn_2_As_12_. The predicted temperature-dependent electrical resistivity and Seebeck coefficient are shown in Figure 9b,c, along with experimentally measured values for *x* = 0.1. The temperature dependency of the resistivity does not match between the DFT calculation where resistivity increases up to 700 K when the carrier concentration is 1 × 10^18^ cm^–3^, and experiments where resistivity decreases with temperature. This is likely due to grain boundaries and other defects, as discussed in more detail below. On the other hand, a comparison of the Seebeck coefficient between DFT calculations and experiments suggests that the carrier concentration of the *x* = 0.1 is between 1 × 10^18^ and 5 × 10^18^ cm^–3^, in agreement with the Hall carrier concentration (Table 1). The anisotropy of hole transport is apparent from the direction-dependent resistivity and Seebeck coefficient (Figure 8d), as expected from the nonspherical Fermi surface (Figure 8). In particular, the electrical resistivity in the *z*-direction is higher than in the *x*- and *y*-directions due to the higher band effective mass in the *z*-direction, as evidenced by the elongation of the tubular Fermi surface geometry in that direction. Directional Seebeck coefficients and resistivities for different carrier concentrations are given in Figure S8 and highlight the fact that Seebeck coefficients depend on the DOS effective mass, which is not directional, while electrical resistivity depends on the band effective mass, which depends on direction. The effective mass from the Seebeck coefficient is calculated from the linear region of the predicted Pisarenko relation at 300 K (Figure 9e) and 500 K (Figure 9f), using the formula in ref (67). We find that the effective mass increases from 0.47*m*_e_ at 300 K to 0.57*m*_e_ at 500 K. The calculated effective mass is slightly higher than the experimental value (determined using the Seebeck coefficient and carrier concentration) of 0.24 ± 0.1*m*_e_ at 300 K for *x* = 0.075 and 0.1.

Both calculated and experimental resistivities decrease with doping, but their temperature dependence differs (Figure 9b). It is important to note that the simulated properties do not address effects on transport properties from grain boundaries, defects, site preference of Na, and commensurate structure modulation. Grain boundary scattering effects often result in a thermally activated decrease in resistivity with temperature, which would be observed especially at lower temperatures.^59^ Since the calculated resistivity increases with temperature at low temperatures and the experimental resistivity decreases with temperature (Figure 9b), grain boundary scattering—as observed in the Hall mobility in Figure 7 discussed above—may cause discrepancies between the DFT predictions and experimental measurements. Grain boundary effects have also been shown to impact Seebeck coefficient in layered structures such as Mg_3_Sb_2_^58,59^ and may explain why experimental Seebeck coefficients increase with doping while theoretical values decrease. Defect chemistry and cation site preference have also been shown to play a major role in electronic transport in layered Zintl phases^15,18,24^ and are not considered in the calculations presented here.

## Summary

Experiment and theory each provide a partial understanding of Eu_11_Zn_4_Sn_2_As_12_, and when used in conjunction, provide deeper insight into this complex, modulated, layered material. Experimental samples are limited by defects, impurities, and microstructure effects, while theory is simplified, using a rigid band model that does not take Na site preference, defects, or the commensurate modulation of the structure into account. Employing both methods together, we get a fuller picture that helps untangle these effects to understand the intrinsic properties of this compound and how defects and microstructure impact thermal and electronic transport. Experimental data reveal low lattice thermal conductivity, high Seebeck coefficient, and electrical resistivity that can be improved through doping, while theoretical results confirm anisotropic transport and a transition to 2D electronic structure when carrier concentration reaches 10^19^ cm^–3^, and the electronic structure becomes more 2D-like with increasing Na content. Experimental results also reveal the complexity of this layered compound and the major influence that microstructure, site preference, and structural disorder have on electronic properties such as the Seebeck coefficient. These results can be used as a roadmap to optimize this promising thermoelectric material by tuning the synthetic method to control microstructure and increasing the carrier concentration to push the Fermi level into the 2D regime.

The unique electronic structure, thermoelectric properties, and a new synthetic method to produce Na-doped Eu_11_Zn_4_Sn_2_As_12_ are presented. Employing a Eu binary precursor such as EuAs allows for high phase purity and reliable reproducibility of this complex structure. Eu_11_Zn_4_Sn_2_As_12_ is a 3D layered Zintl phase that exhibits a 2D electronic structure at carrier concentrations greater than 1.5 × 10^19^ cm^–3^ due to anisotropic bonding between covalent nets and ionic layers. Experimental measurements are consistent with a complex, nonparabolic band structure. These results offer an initial understanding of a complicated system and guide further improvement of this material. Additional substitution on the Zn, Sn, or As sites could take advantage of the 2D electronic transport, dramatically increasing *zT* and making this material promising for various applications. This study also shows that directional bonding in layered Zintl phases with 3D crystal structures gives rise to low-dimensional electronic structures, providing a new direction for research on these compounds.

## Supporting Information Available

Rietveld refinements of EuAs and Eu_11-*x*_Na_*x*_Zn_4_Sn_2_As_1_ (*x* = 0, 0.05, 0.075, 0.1), Rietveld refinement statistics for Eu_11-*x*_Na_*x*_Zn_4_Sn_2_As_12_, Rietveld refinement of a pellet, quantitative EDS table, SEM micrographs and EDS elemental maps, Hall carrier concentrations (300 K), XPS data of As and Eu 3*d* regions after 10 and 180 min Ar sputtering, simulated directional Seebeck coefficients and electrical resistivities, and experimental *zTs* (PDF)
