# Supplementary material for: Experiment and Theory in Concert To Unravel the Remarkable Electronic Properties of Na-Doped Eu11Zn4Sn2As12: A Layered Zintl Phase
Source: Chem Mater. 2023 Sep 14;35(18):7719–29. doi: 10.1021/acs.chemmater.3c01509 (PMC10536979; doi:10.1021/acs.chemmater.3c01509)
Supplement: Supplementary file 1 — cm3c01509_si_001.pdf [file cm3c01509_si_001.pdf]

Experiment and Theory in Concert To Unravel the Remarkable Electronic Properties of Na-Doped $Eu_{11}Zn_4Sn_2As_{12}$: A Layered Zintl Phase

Ashlee K. Hauble[1], Michael Y. Toriyama[2], Stephan Bartling,[3] Ali M. Abdel-Mageed,[3] G. Jeffrey Snyder[2*], and Susan M. Kauzlarich[1*]

Affiliations

[1]Department of Chemistry, One Shields Ave, University of California, Davis, California 95616, United States
[2]Department of Materials Science and Engineering, Northwestern University, Evanston, IL 60208, United States
[3]Leibniz Institute for Catalysis (LIKAT), Rostock 18059, Germany

*Corresponding author's emails: jeff.snyder@northwestern.edu & smkauzlarich@ucdavis.edu

Supporting Information

Table of Contents

Table S1. Statistics for Rietveld refinements of EuAs PXRD data

Figure S1. Rietveld refinement of EuAs PXRD data

Figure S2. Rietveld refinements of $Eu_{11-x}Na_xZn_4Sn_2As_{12}$ (x = 0, 0.05) PXRD data

Figure S3. Rietveld refinements of $Eu_{11-x}Na_xZn_4Sn_2As_{12}$ (x = 0.075, 0.1) PXRD data

Table S2. Statistics for Rietveld refinements of $Eu_{11-x}Na_xZn_4Sn_2As_{12}$ (x = 0, 0.05, 0.075, 0.1) PXRD data

Figure S4. Rietveld refinement of a pressed pellet of $Eu_{11}Zn_4Sn_2As_{12}$

Table S3. EDS results on pellets of $Eu_{11-x}Na_xZn_4Sn_2As_{12}$ compared to nominal compositions

Figure S5. SEM micrographs and EDS elemental maps

Table S4. Hall carrier concentration at 300 K

Figure S6. XP spectra of As and Eu 3*d* region after 10 minutes Ar sputtering

Figure S7. XP spectra of As and Eu 3*d* region after 180 minutes Ar sputtering

Figure S8. Simulated directional Seebeck coefficients and electrical resistivity

Figure S9. Temperature dependent *zT*s

Table S1. Selected Rietveld Refinement Statistics for a Typical Powder X-ray Diffraction Pattern of EuAs (ICSD- 26265, the Na_2O_2 structure type)

**x**		**EuAs**
**Unit Cell Parameters**	*a*	8.158(1)
	b	6.140(1)
	V	353.92(1)
**Rp (%), Rwp (%)**	11.01, 12.08	
**EuAs (wt %)**	97.02	
**Eu_3As_4 (wt %)**	2.98	

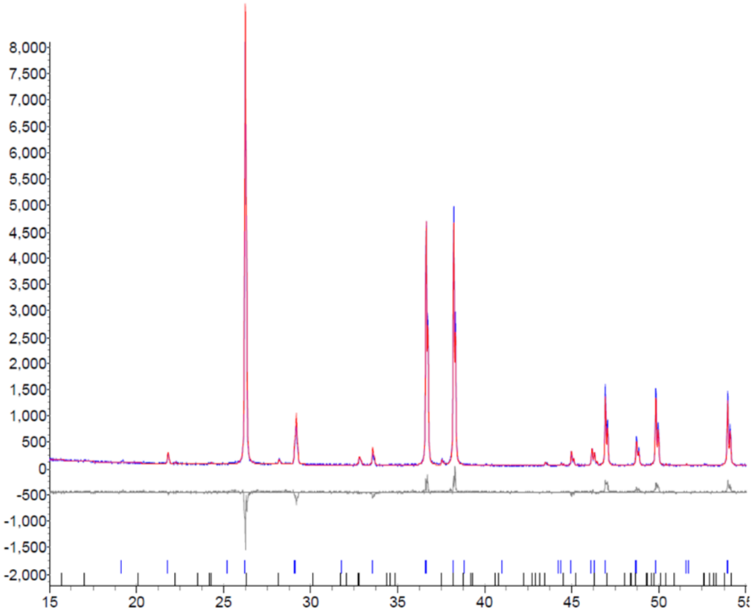

Figure S1. A portion of typical Rietveld refinement for EuAs (ICSD- 26265, the Na_2O_2 structure type). Observed data are shown in black, calculated pattern in red, and difference curve in gray. Tick marks indicate EuAs (blue) and Eu_3As_4 (black).

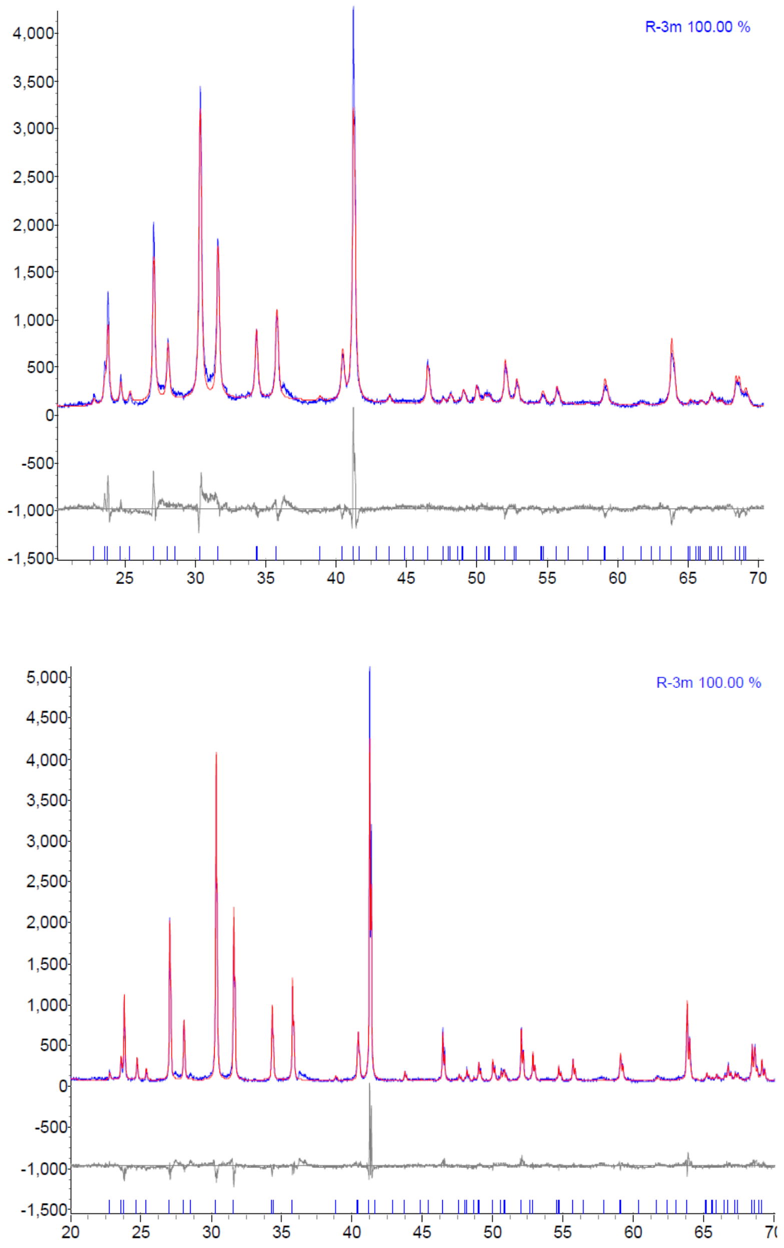

Figure S2. A portion of a typical Rietveld refinement for $Eu_{11}Zn_4Sn_2As_{12}$ (top) and $Eu_{10.95}Na_{0.05}Zn_4Sn_2As_{12}$ (bottom) was prepared from EuAs. Observed data are shown in black, the calculated pattern in red, and the difference curve in gray.

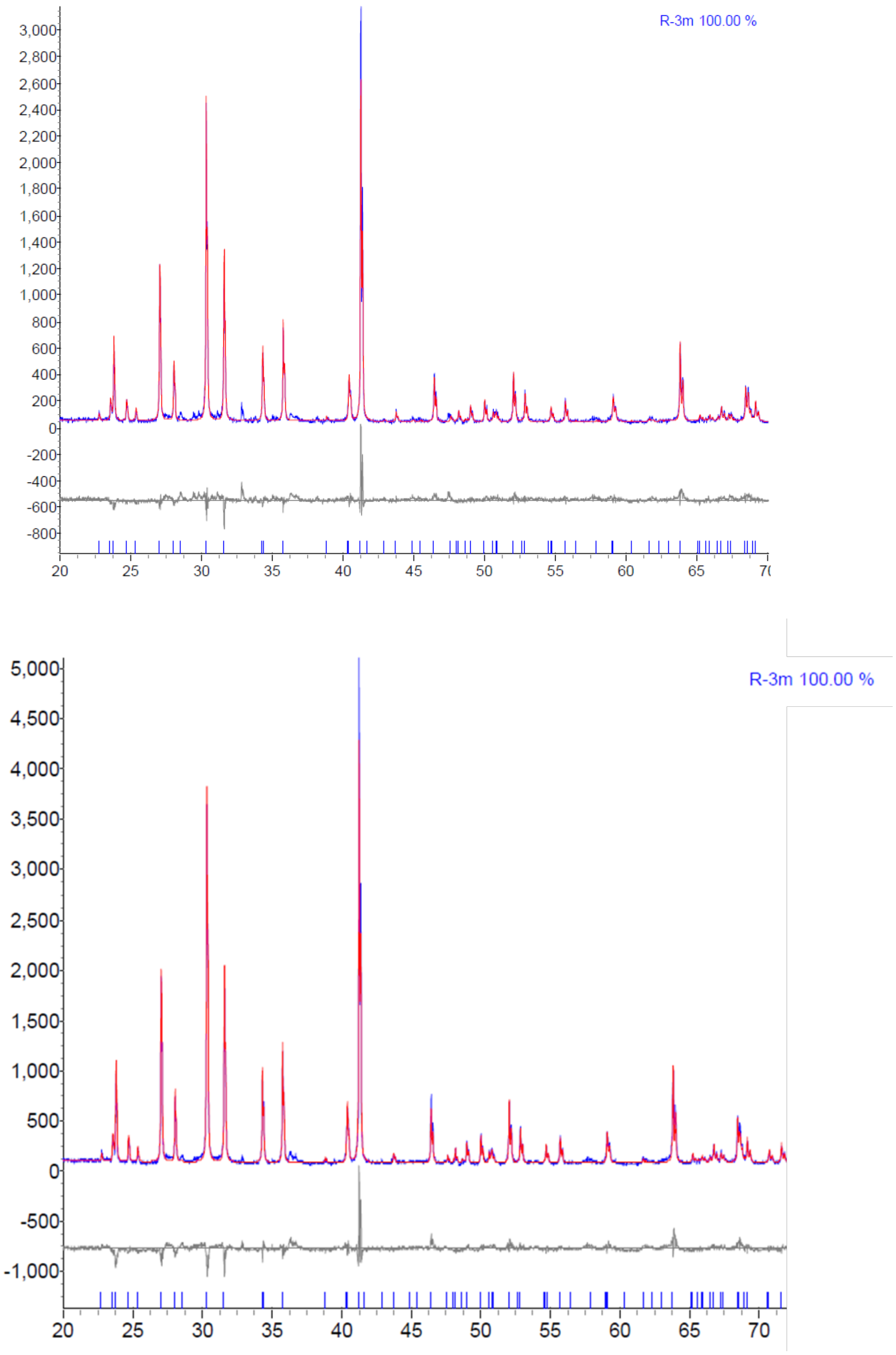

Figure S3. A portion of a typical Rietveld refinement for $Eu_{10.925}Na_{0.075}Zn_4Sn_2As_{12}$ (top) and $Eu_{10.9}Na_{0.1}Zn_4Sn_2As_{12}$ (bottom) made from EuAs. Observed data are shown in black, the calculated pattern in red, and the difference curve in gray.

Table S2. Selected Rietveld Refinement Statistics for $Eu_{11-x}Na_xZn_4Sn_2As_{12}$ (x = 0, 0.05, 0.075, 0.1).

x		**0**	**0.05**	**0.075**	**0.1**
**Unit Cell Parameters** $R\bar{3}m$	a (Å)	4.3794(2)	4.37664(9)	4.3768(1)	4.37620(7)
	c (Å)	46.865(3)	46.912(1)	46.921(1)	46.935(9)
	V (Å^3)	778.41(4)	778.21(4)	778.43(4)	778.43(3)
**($R\bar{3}m$) Rp (%), Rwp (%)**		11.30, 13.96	11.47, 14.73	12.92, 16.88	11.92, 14.92
**($R\bar{3}$) Rp (%), Rwp (%)**		13.88, 17.92	11.34, 14.54	12.72, 16.63	11.50, 14.56
**($C2/c$) Rp (%), Rwp (%)**		11.53, 14.83	12.61,18.02	14.10, 19.76	11.24, 16.14
**$Eu_{11}Zn_4Sn_2As_{12}$ (wt %)**		100	100	100	100

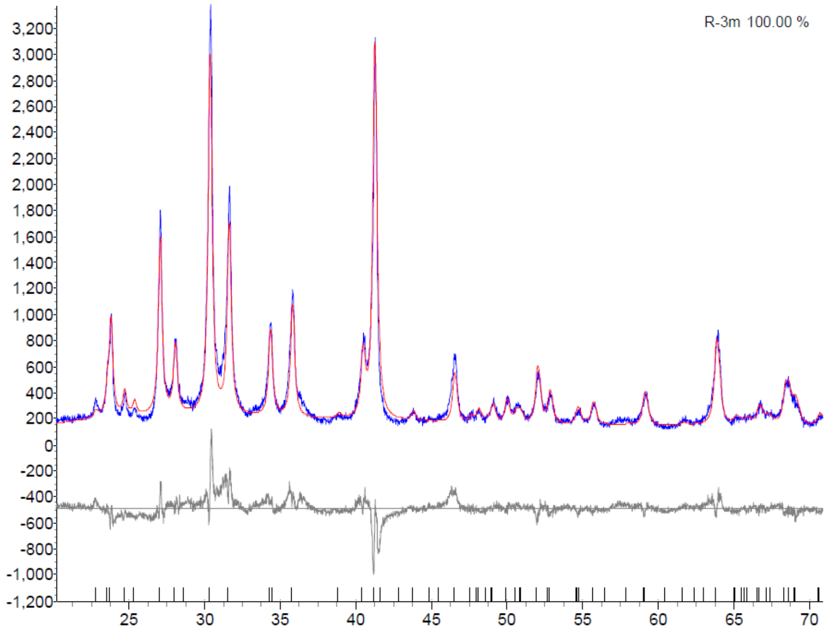

Figure S4. A portion of a typical Rietveld refinement for a pressed pellet of $Eu_{11}Zn_4Sn_2As_{12}$ made from EuAs. Observed data are shown in black, the calculated pattern in red, and the difference curve in gray. No preferred orientation is observed.

STable 3. Energy Dispersive Spectroscopy (EDS)[1] on Pellets of $Eu_{11-x}Na_xZn_4Sn_2As_{12}$ Compared to Nominal Compositions

x	Eu %	Eu nom. %	Zn %	Zn nom. %	Sn %	Sn nom. %	As %	As nom. %
0	39.9(6)	37.93	13 (2)	13.79	6.54(8)	6.90	40.3(3)	41.38
0.05	39.6(9)	37.76	13 (1)	13.79	6.72(7)	6.90	40.0(2)	41.38
0.075	36.1(8)	37.67	12.4(2)	13.79	7.07(6)	6.90	44.4(3)	41.38
0.1	39.1(9)	37.58	14.5(4)	13.79	6.74(4)	6.90	39.7(2)	41.38

[1] based on 10 data points randomly placed on each pellet

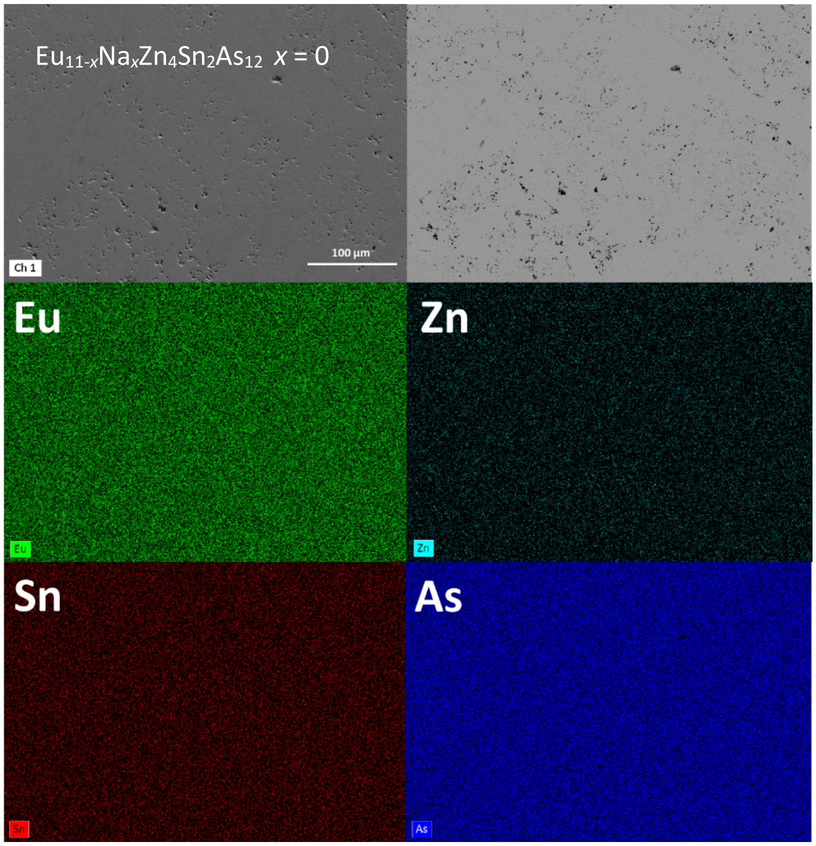
Eu11-xNaxZn4Sn2As12 x = 0
Ch 1
100 µm
Eu
Zn
Sn
As

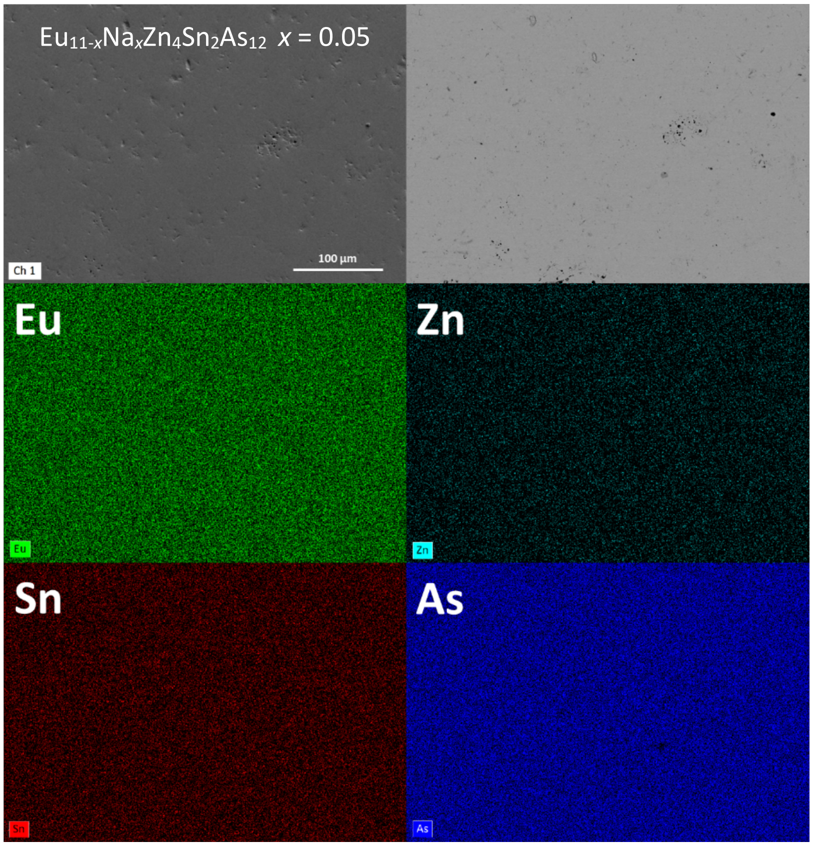
Eu11-xNaxZn4Sn2As12 x = 0.05
Ch 1
100 µm
Eu
Zn
Sn
As

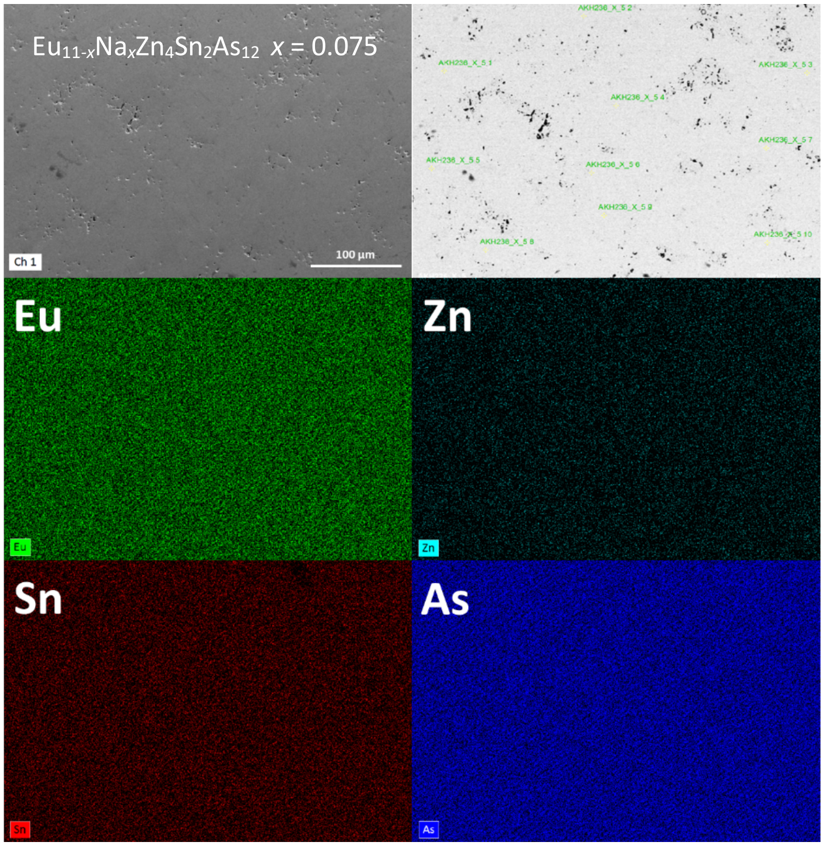

Figure S5. Secondary electron SEM micrographs (top left), backscattered electron SEM micrographs (top right) and EDS elemental maps of $Eu_{11-x}Na_xZn_4Sn_2As_{12}$ for x = 0 (top), 0.05 (middle) and 0.075 (bottom).

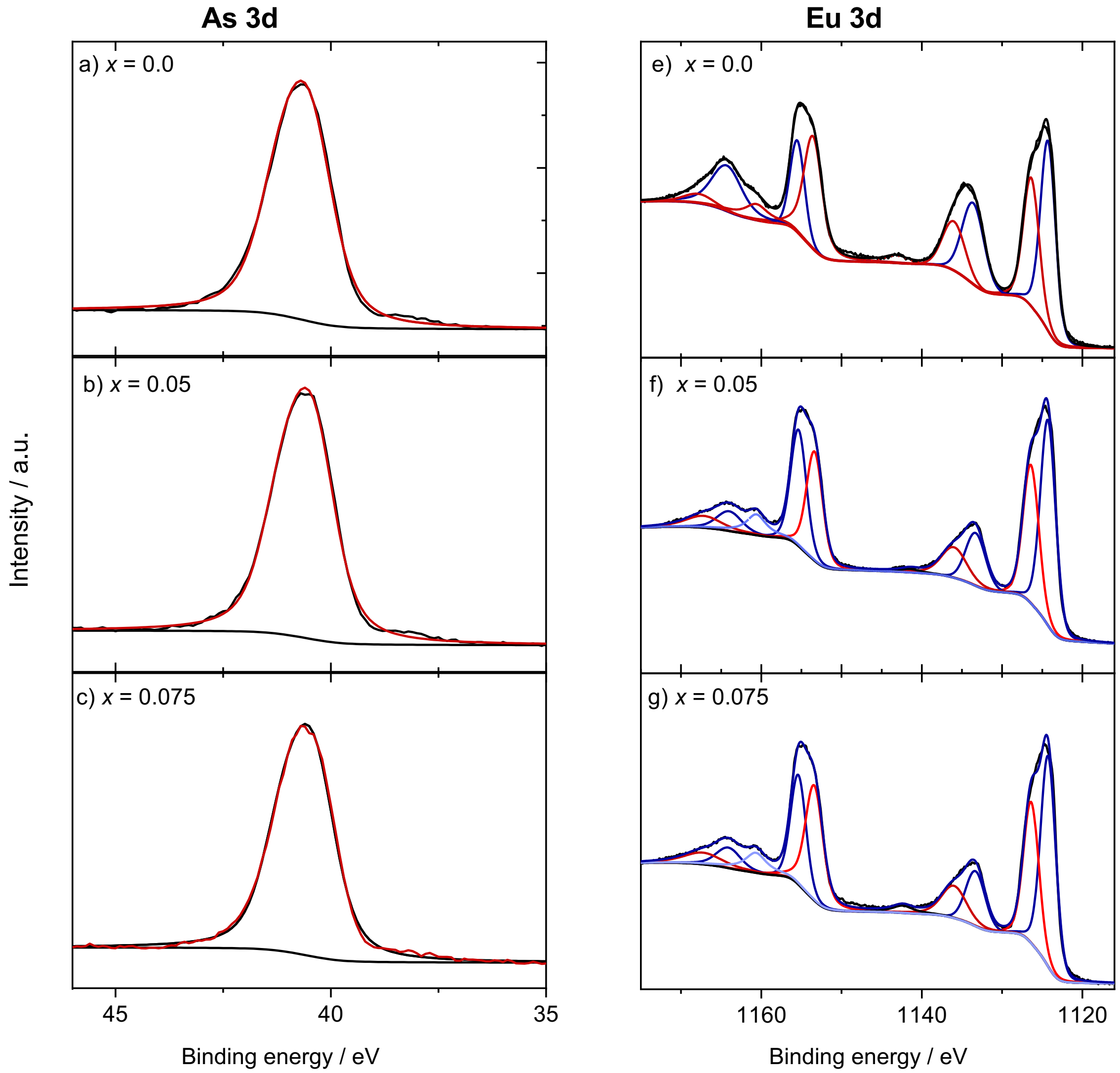

Figure S6. XP spectra in the As and the Eu 3*d* (a: *x* = 0; b: *x* = 0.05 ; c: *x* = 0.075; d: *x* = 0.1) region. Measurement is taken after 10 min of Ar sputtering.

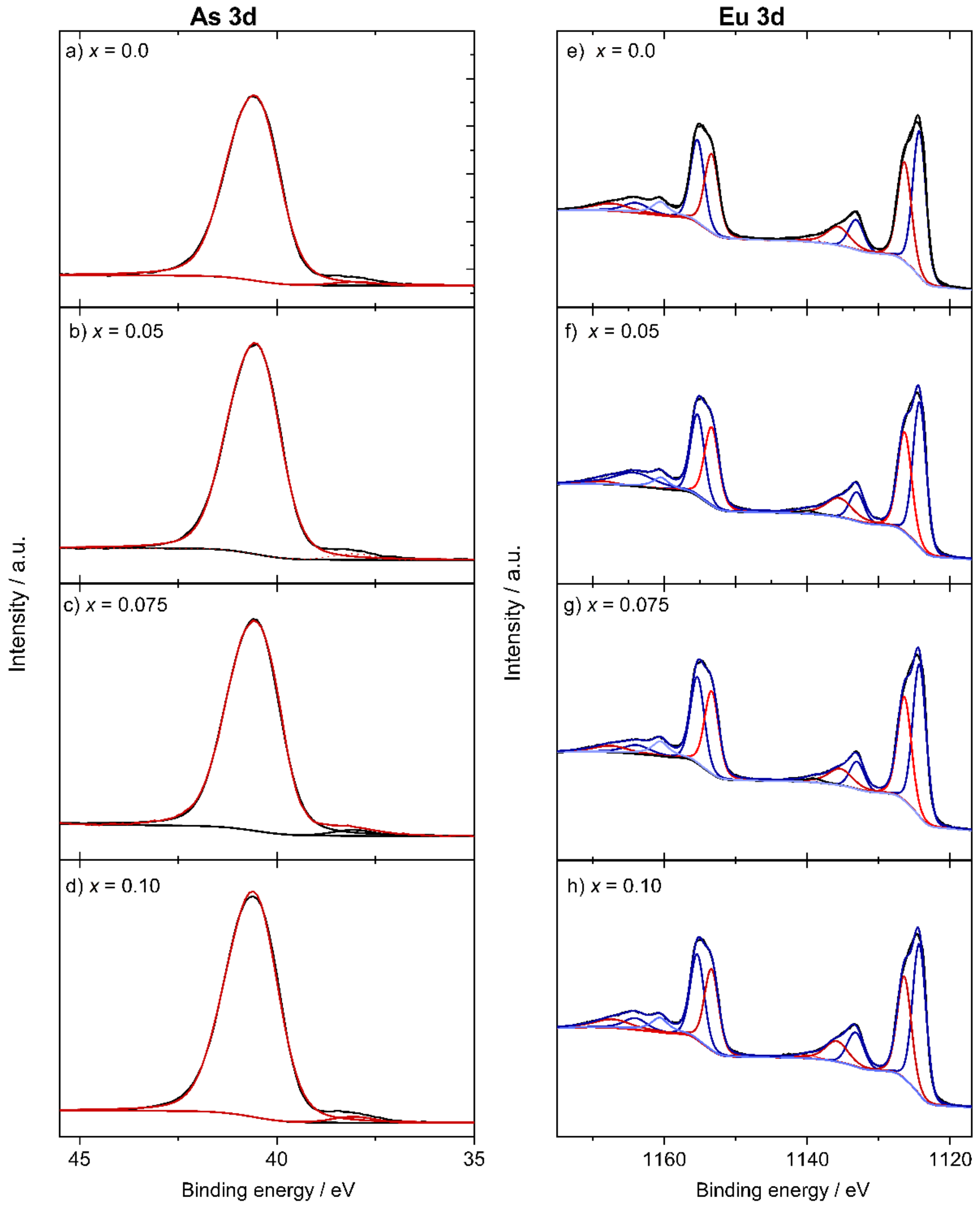

Figure S7. XP spectra in the As and the Eu 3*d* (a: x = 0; b: x = 0.05 ; c: x = 0.075; d: x = 0.1) region. Measurement was taken after 180 min of Ar sputtering.

Table S4. Hall Carrier Concentrations for $Eu_{11-x}Na_xZn_4Sn_2As_{12}$

x	**Carrier Concentration (cm^{-3})** **300 K**
0	4.01×10^{17}
0.05	4.52×10^{17}
0.075	1.04×10^{18}
0.1	1.14×10^{18}

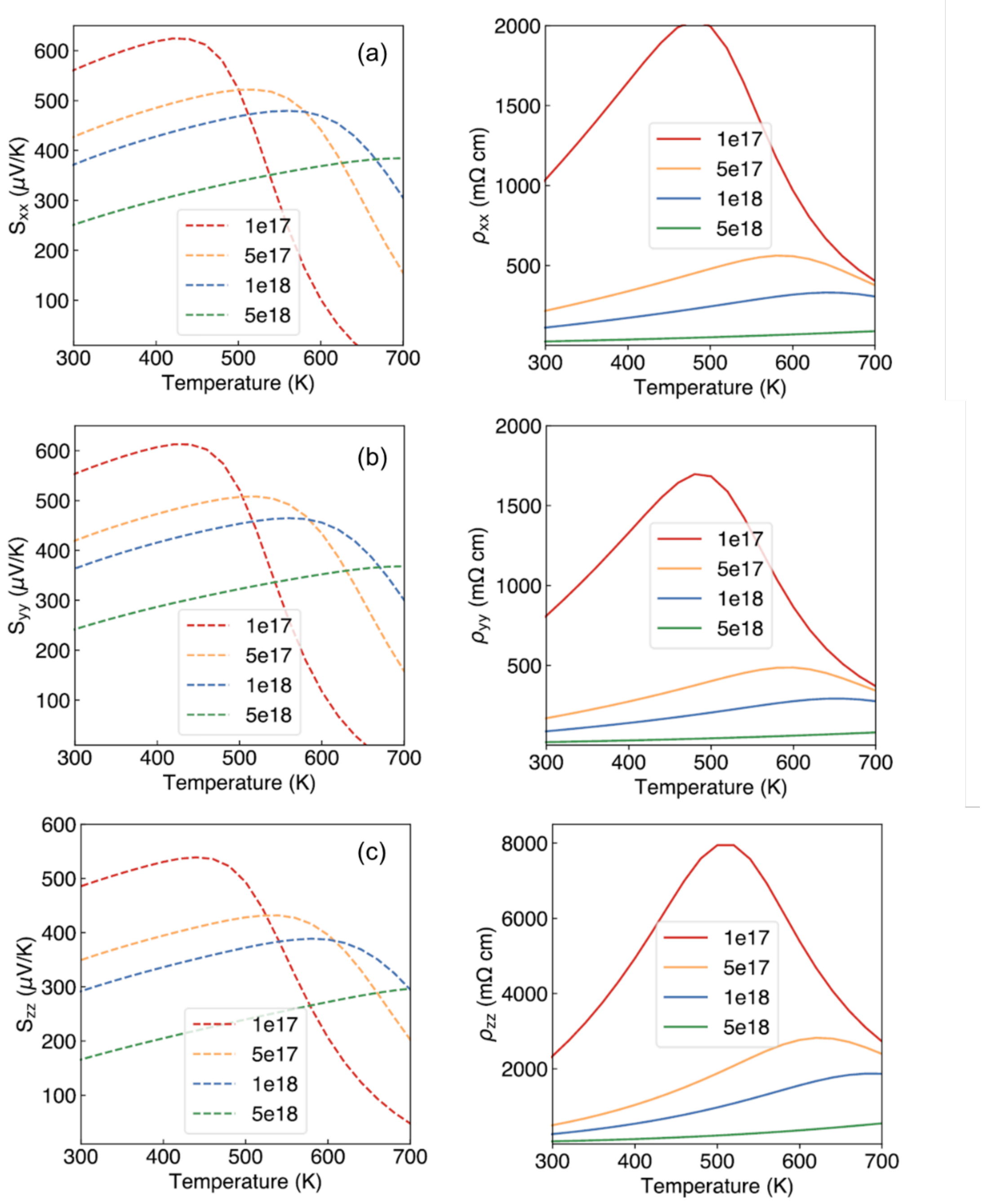

Figure S8. Simulated directional Seebek coefficient and electrical resistivity for varying carrier concentrations. Note that Seebeck coefficient and electrical resistivity values are similar for (a) x and (b) directions, while Seebeck coefficients are lower and electrical resistivity is higher in (c) the z direction.

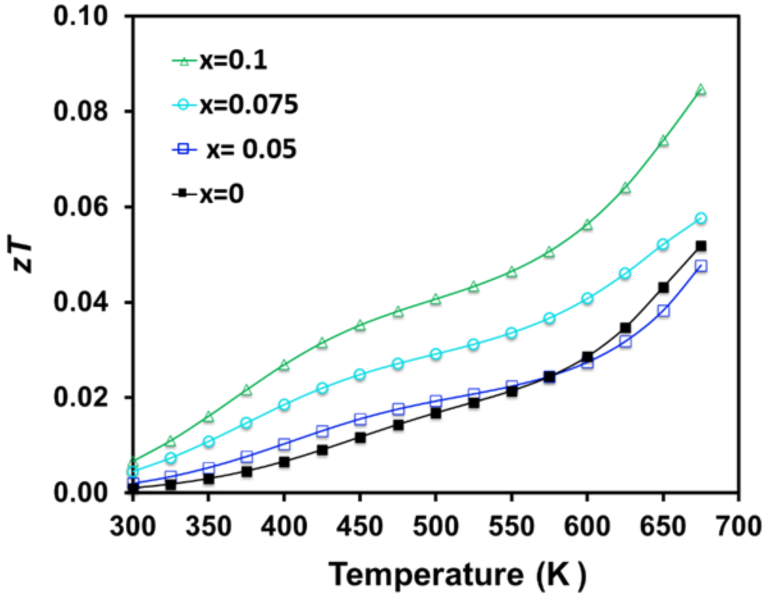

Figure S9. Temperature dependent *zT*s for $Eu_{11-x}Na_xZn_4Sn_2As_{12}$ (x = 0, 0.05, 0.075, 0.1).
